# Bioactivity-Guided Separation of Potential D_2_ Dopamine Receptor Antagonists from Aurantii Fructus based on Molecular Docking Combined with High-Speed Counter-Current Chromatography

**DOI:** 10.3390/molecules23123135

**Published:** 2018-11-29

**Authors:** Yingjie He, Shihao Zhu, Changqiao Wu, Ying Lu, Qi Tang

**Affiliations:** 1 Hunan Key Laboratory of Traditional Chinese Veterinary Medicine, Hunan Agricultural University, Changsha 410128, China; yingjiehe272@163.com (Y.H.); zsh190305763@163.com (S.Z.); m15207494911@163.com (C.W.); 2 Horticulture and Landscape College, Hunan Agricultural University, Changsha 410128, China

**Keywords:** Aurantii fructus, molecular docking, high-speed counter-current chromatography, D_2_ dopamine receptor antagonists, naringenin

## Abstract

The typical compounds of Aurantii fructus (AF) reported in previous research were screened for their high antagonistic ability on the D_2_ dopamine receptor (D_2_R) in silico, and then bioactivity-guided separation was undertaken on the potential D_2_R antagonists from AF using high-speed counter-current chromatography (HSCCC). Three flavanones, two polymethoxyflavonoids, and three coumarins were effectively isolated from ethanol extracts of Aurantii fructus (AF) by the use of a two-step HSCCC method, and their chemical structures were identified by mass spectrometry, ^1^H-NMR, and ^13^C-NMR and compared with published data. Firstly, crude extract of 70% ethanol eluent (150 mg) was isolated by HSCCC using an *n*-hexane−ethyl acetate−*n*-butanol−methanol−0.05% acetic acid (1:3:1.8:1:5, *v*/*v*/*v*/*v*/*v*) solvent system, and compounds **1** (naringin, 28 mg), **2** (neohesperidin, 13 mg), **3** (meranzin, 5 mg) and **4** (poncirin, 3 mg) were successfully isolated with 98.5%, 95.1%, 97.7%, and 92.4% purity, respectively. Then, the crude extract of 95% ethanol eluent (120 mg) was isolated by *n*-hexane−*n*-butanol−ethanol (methanol)−0.05% acetic acid (2:0.6:1:3, *v*/*v*/*v*/*v*) solvent system and compounds **3** (meranzin, 3 mg), **5** (meranzin hydrate, 4 mg), **6** (isomeranzin, 6 mg), **7** (nobiletin, 10 mg), and **8** (tangeretin, 7 mg) were successfully isolated with 95.8%, 98.5%, 95.1%, 92.4%, and 97.7% purity, respectively. Naringenin, a parent structure of naringin with the excellent binding score of −9.3 kcal/mol, was completely in conjunction with the active site of D_2_R, indicating that it is critical for the treatment of gastrointestinal dysfunction. The results indicated that the bioactivity-guided method is practical for the effective separation of active compounds from natural resources.

## 1. Introduction

As a traditional Chinese medicine, the use of Aurantii fructus (AF) is mainly focused on the treatment of gastrointestinal dysfunction, improvement of qi stagnation, and remission of chest pain in traditional therapies, and it is sourced from the green immature fruit of *Citrus aurantium* L. [1]. The clinical pharmacological research of previous studies has showed that the secondary metabolites (e.g., flavonoids and coumarins) are considered as the main bioactive compounds with enriched content, and play an important role in the regulation of gastrointestinal disorders [2]. However, the pharmacological mechanism of its action on specific targets has not been illustrated.

In the gastrointestinal tract, D_2_ dopamine receptors (D_2_Rs) are the main receptors found on the gastrointestinal smooth muscles (GSMs) to regulate the contraction and relaxation of GSMs [3]. D_2_R antagonists such as domperidone can reduce the dopamine-mediated relaxation of gastric smooth muscles and promote gastric motility, and are the most popular medicines to promote gastrointestinal motility [4]. Docking simulations can screen bioactive D_2_R antagonists and it is meaningful to screen for the safety, efficacy, and low side-effects of D_2_R antagonists from traditional medicines that have been widely recognized as important sources of bioactive compounds with remarkable effects and health benefits. Besides this, the clinical pharmacological mechanism of small molecules on target receptors for the treatment of gastrointestinal motility disorder could also be illustrated on a molecular level. Therefore, based on the reported data and the basic chemical substances identified and speculated in our previous study on AF [2], these typical bioactive compounds capable of regulating gastrointestinal motility disorders could be further discussed and preliminarily screened out by molecular docking simulation analysis in silico [5]. Additionally, the screening of D_2_R antagonists from AF using molecular docking could be an efficient bioactivity guide for the further separation of active compounds.

The conventional separation methods for complicated extracts of herbs generally require multiple separation and elution steps on macroporous resin, silica gel, polyamide, thin-layer, etc., and a number of the common methods mentioned above are utilized for isolating chemical compounds from AF [6,7]. Although many components have been successfully isolated from AF by these methods, there are many drawbacks to the use of these techniques for this application due to the similarities of the compounds’ chemical structures, polarities, and labilities. There are also other limitations regarding of these techniques, such as the irreversible adsorption of target compounds, their complicated isolation steps, and their time-consuming nature, high-cost, large solvent consumption, and secondary pollution [8]. Furthermore, these methods have a low ability for the large-scale isolation and purification of active compounds from AF. To avoid these disadvantages, high-speed counter-current chromatography (HSCCC), a durative method of liquid–liquid partition, has been employed and developed to separate and purify valuable bioactive constituents from herbs and other natural sources because of its particular performance in isolating target components with concise steps, high recovery, and non-irreversible adsorptions [9,10]. In addition, HSCCC is considered as an adequate preparative tool for the large-scale isolation and purification of target ingredients through sufficient interaction between the liquid-stationary phase and the analytes.

Many reports have presented the tentative identification and isolation of flavonoid and coumarin compounds from citrus plants. However, to date, there are few reports on the HSCCC preparation of these multiple compounds from AF [7], the bioactivity-guided separation of D_2_R antagonists from natural resources has not been reported, and it is still a complicated and challenging task. Therefore, in the present study, a novel method of bioactivity-guided two-step HSCCC separation was established to purify a series of active compounds with high purity by the use of two solvent systems: *n*-hexane−ethyl acetate−*n*-butanol−methanol−0.05% acetic acid (1:3:1.8:1:5, *v*/*v*/*v*/*v*/*v*) and *n*-hexane−*n*-butanol−ethanol (methanol)−0.05% acetic acid (2:0.6:1:3, *v*/*v*/*v*/*v*). In addition, the molecular mechanism of AF’s action in the treatment of gastrointestinal dysfunction was illustrated by molecular docking research.

## 2. Results and Discussion

### 2.1. Screening of Potential D_2_R Antagonists

In drug discovery and development, molecular docking simulation is commonly employed for the screening of potential and specific active compounds, including the discovery of potential D_2_R antagonists. As one of the best screening tools, Autodock is very powerful in its ability to estimate the binding affinity between specific ligands and proteins. In the present study, a molecular docking simulation was developed to simulate the molecular recognition process between D_2_R and the secondary metabolites of AF, and the binding affinities were evaluated and calculated simultaneously as shown in Table S1. In general, it is important to note that the docking score should be close to or over 80% as compared to the positive compound [11]. In addition, for practical application production, the screened compound should be isolated at preparative scale. As a result, a series of compounds were preliminarily screened from AF according to their individual docking score, potential prediction, and feasibility of preparative separation as shown in Table 1. The excellent compounds with high scores and feasible separation were mainly flavonoids (e.g., flavanones and polymethoxyflavonoids) and coumarins. Therefore, these active compounds (e.g., naringenin, tangeretin, meranzin hydrate, and other structurally similar compounds) should be further isolated. Before entering the body, chemical compounds are hydrolyzed to metabolites in the gastrointestinal tract and then absorbed for their corresponding pharmacological effects [12]. Flavonoid glycosides of AF such as naringin, neohesperidin, and poncirin were hydrolyzed to naringenin, hesperetin, isosakuranetin, etc. Therefore, in the screening process, attention should be paid to the relationship between glycosides and their prototypes. Especially for naringenin, with the best docking score, the preparative separation of its flavonoid glycosides (naringin) is very meaningful.

**Figure 1:**
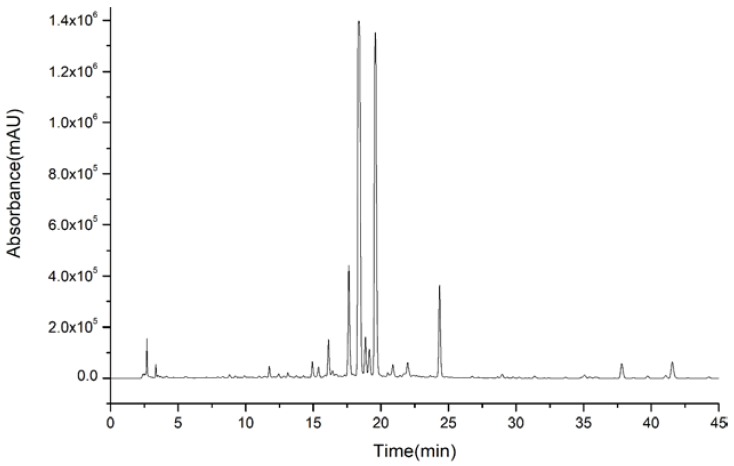
High performance liquid chromatography (HPLC) of ethanol extract of Aurantii fructus.

Therefore, the next separation procedures were concentrated on these typical compounds. Combined with our previous study on the basic components of AF, the chemical compounds of AF ethanol extract were qualitatively analyzed by LC-Q-TOF-MS and presented a complicated composition with varied polarity, as shown in Figure 1. The chromatographic peaks of flavanones and coumarins intensively appeared from 10 to 36 min, and the chromatographic peaks of polymethoxyflavonoids mainly appeared after 36 min. According to the difference of polarity distribution, the target compounds were assigned and prepared for the next separation.

### 2.2. Preparation of Ethanol Extracts

**Figure 2:**
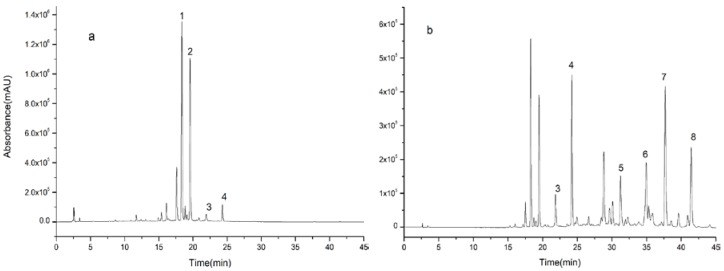
High performance liquid chromatography (HPLC) of ethanol elution of Aurantii fructus by the use of D101 macroporous resin, (**a**) 70% ethanol eluent and (**b**) 95% ethanol eluent.

D101 macroporous resin is commonly used to separate and purify natural product [7]. Firstly, it can remove non-adsorbent components such as polysaccharides and proteins from extracts. On the other hand, they can separate compounds by gradient elution and enrich some low-content components. Experiments showed that D101 macroporous resin could separate and enrich chemical compounds from AF. The elution effect of ethanol at different concentrations is shown in Figure 2. It can be seen in Figure 2a that the chromatographic peaks in 70% ethanol eluent mainly appeared before 25 min, and the chromatographic peaks after 25 min were all in the 95% ethanol eluent and were effectively enriched as shown in Figure 2b.

### 2.3. Optimization of HSCCC

HSCCC is an efficient instrument for the isolation of chemical compounds from natural sources. The two-phase solvent system, flow rate, temperature, and rotation rate are essential factors that can impact the productivity of the HSCCC purification. For example, the solvent system affects the *K*-values of compounds that are closely related with purity, low flow rate generates good separation, but requires a long isolation time and more mobile phase organic solvent, and the speed of revolution and the temperature affect the retention rate of stationary phase [13]. Therefore, referring to the reported literatures [14,15] and combining with our previously optimal separation conditions, the multilayered coils were first entirely filled with the upper stationary phase at a flow rate of 5.0 mL/min, and the flow rate of the mobile phase (2.0 mL/min), the temperature (20 °C), and the rotation rate (900 rpm) were determined for the optimum isolation.

### 2.4. Two-Phase Solvent Systems

The choice of an appropriate two-phase solvent system is essential for efficient isolation in HSCCC [16]. An efficient HSCCC isolation usually requires the selection of a suitable solvent system with favorable partition coefficients (*K*-values) for the target ingredients [15]. In general, ideal *K*-values are expected between 0.5–2.0 [17]. According to the structural characteristics of these target AF compounds (flavonoids and coumarins), a series of solvent systems containing *n*-hexane/ethyl acetate/*n*-butanol/methanol/ethanol/acetic acid/water were used for partition coefficient tests.

### 2.5. Separations of Compounds ***1***, ***2***, ***3***, and ***4***

**Figure 3:**
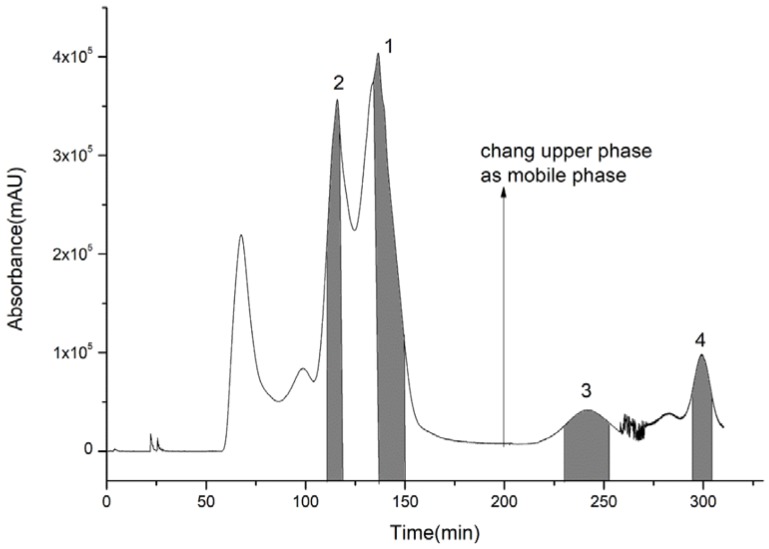
Extract of 70% ethanol eluent via high-speed counter-current chromatography.

**Figure 4:**
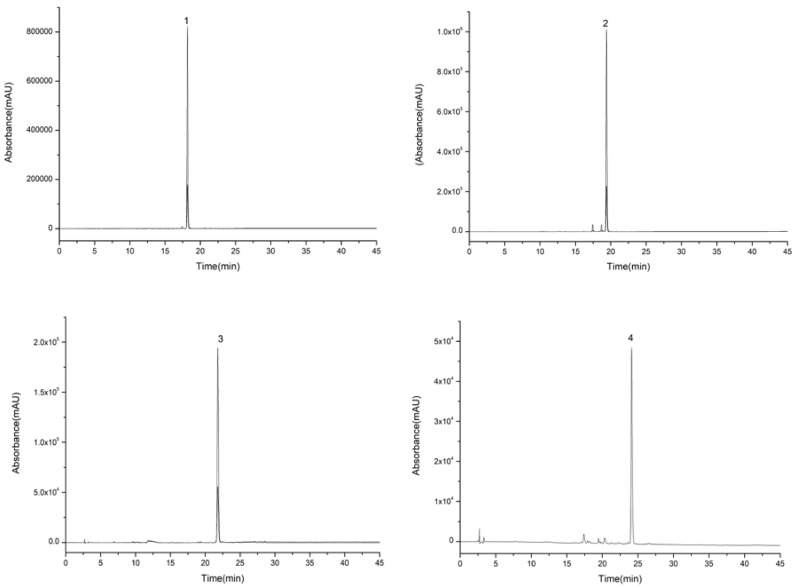
High performance liquid chromatography graphs of compounds (**1**, **2**, **3**, and **4**) eluted from extract of 70% ethanol eluent.

For the first step of HSCCC isolation, a series of solvent systems at varied proportions were tested. The *K*-values of four target compounds were tested via HPLC analysis as shown in Table 2. The *K*-values of the four compounds were ideal between 0.5 and 5 in Systems 3 and 4. However, when System 4 was performed, the purity of compounds **1** and **2** was lower than that of System 3. System 3 was adopted for isolation, and the retention rate of the upper phase was close to 64%. It was still found that compound **4** could not be eluted after System 3 was operated at the flow speed of 2.0 mL/min for 350 min. Therefore, the pump was stopped when the lower phase ran for about 200 min, and then the upper phase was replaced as the mobile phase. Because the solubility of compounds **3** and **4** in the upper phase was high, and *K*3 was higher than *K*4, compound **3** eluted before compound **4**, and the operation was controlled in 320 min which greatly shortened the preparation time and reduced the amount of solvents. Compounds **1** (130–150 min), **2** (110–118 min), **3** (230–251 min), and **4** (295–303 min) were collected according to the peak shapes as shown in Figure 3. The collected fractions were condensed and dried, and then the contents of compounds **1**, **2**, **3**, and **4** were calculated by area normalization at 28, 13, 5, and 3 mg with 98.5%, 95.1%, 97.7%, and 92.4% purity, respectively (Figure 4).

### 2.6. Separations of Compounds ***5***, ***6***, ***7***, and ***8***

**Figure 5:**
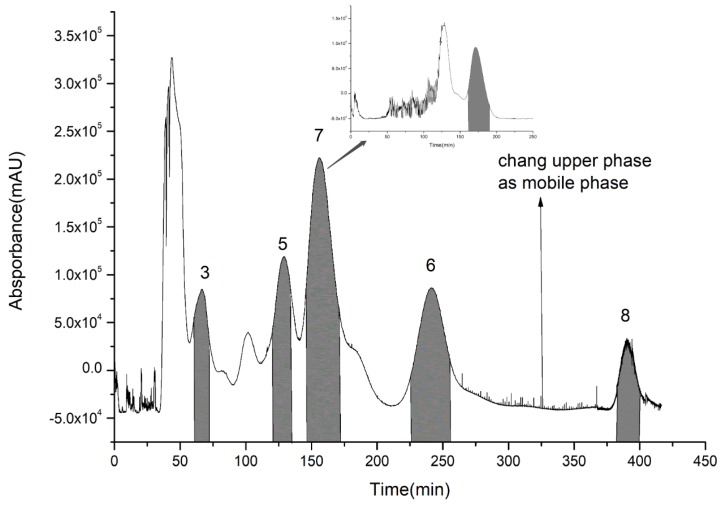
Extract of 95% ethanol eluent via high-speed counter-current chromatography.

**Figure 6:**
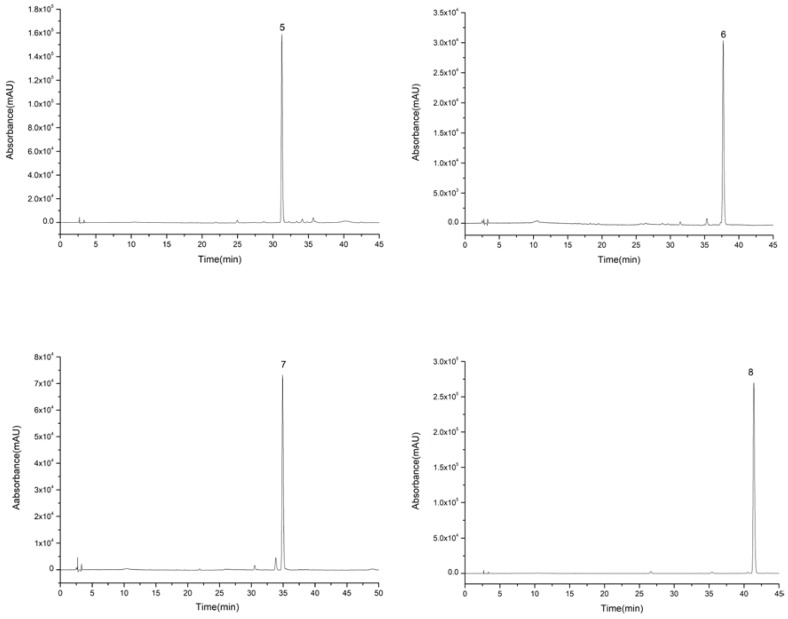
High performance liquid chromatography graphs of compounds **5**–**8** eluted from extract of 95% ethanol eluent.

For the second step of HSCCC isolation, the HSCCC solvent systems were screened by *K*-value determination as shown in Table 3. In the systems consisting of *n*-hexane, *n*-butanol, methanol (ethanol), and water, *K*-values of compounds **5**–**8** were suitable. However, compounds **7** and **8** with high purity could only be obtained from System 8, while compounds **3**, **5**, **6**, and **8** with high purity could be obtained from System 7. Therefore, compounds **3**, **5**, **6**, **7**, and **8** were isolated from System 7, and then compound **7** was further purified by System 8. As in the first step, because of the high *K*-value of compound **8**, the upper phase was changed into the mobile phase at about 320 min. Compounds **3** (63–74 min), **5** (122–132 min), **7** (148–174 min), **6** (226–253 min), and **8** (383–400 min) were collected according to their chromatographic peak shapes and lyophilized as shown in Figure 5. Then, compound **7** was further purified by System 8. The contents of compounds **3**, **5**, **6**, **7**, and **8** were calculated by area normalization method at 3, 4, 5, 10, and 7 mg with 95.8%, 98.5%, 95.1%, 92.4%, and 97.7% purity, respectively (Figure 6).

### 2.7. Structure Identification

**Figure 7:**
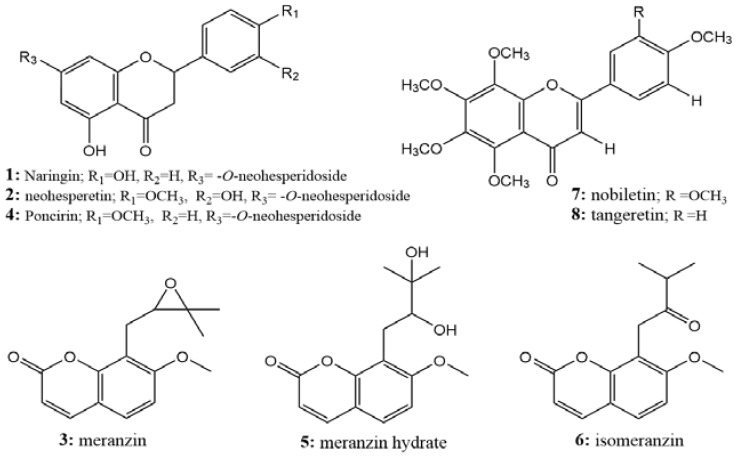
Structures of the isolated compounds.

All of the isolated compounds with high purity were identified by ESI-Q-TOF-MS, ^1^H-NMR, ^13^C-NMR, and standards, and their detailed data are shown in Figure S1. Compounds **1**, **2**, and **4** were identified as naringin [18,19], neohesperidin [19], and poncirin [20], respectively, which belong to the flavanones; compounds **3**, **5**, and **6** were identified as meranzin [18], meranzin hydrate [21], and isomeranzin [18], which belong to the coumarins; compounds **7** and **8** were identified as nobiletin [6,21], and tangeretin [21,22] and belong to the polymethoxyflavonoids (Figure 7). The potential targets of D_2_R antagonists, such as naringin (naringenin), meranzin hydrate, and tangeretin, were successfully guided and separated from AF. This was the first time that multiple compounds were separated simultaneously from AF by a method that could be used in the practical production of separation. Moreover, among these separated compounds, poncirin, meranzin, and isomeranzin were firstly purified at large-scale from AF by HSCCC.

### 2.8. Illustration of Molecular Mechanism of Active Compounds on D_2_R

The antagonistic effect of D_2_R is generated by the interaction between antagonist and residues of the bioactive site (TRP-100, PHE-110, VAL-115, ALA-122, PHE-198, PHE-382, TRP-386, PHE-389, PHE-390, ASP-114) within a range of 4 Å, and interact by hydrophobic and hydrogen bonding interactions [23], etc. The docking results (Table S1) showed that the binding energies of these compounds ranged from −3.5 to −9.3 kcal/mol. Among these binding scores, naringenin had the lowest binding energy (−9.3 kcal/mol) and the molecular mechanism of naringenin to D_2_R was consistent with the positive drug (domperidone and risperidone) as shown in Table 4.

**Figure 8:**
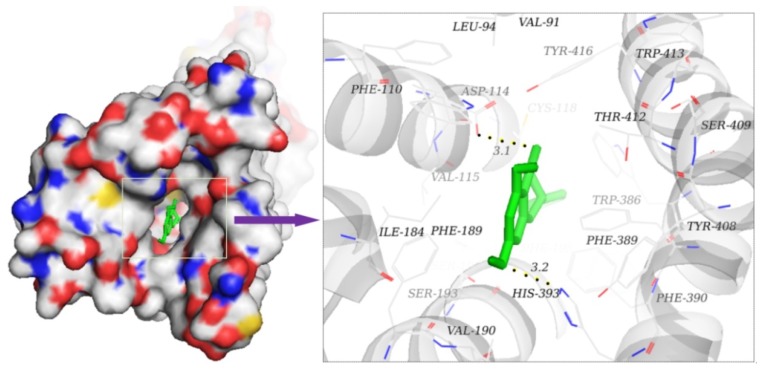
Binding mode prediction of naringenin to D_2_R via AutoDock. Naringenin is shown in green stick form and black dashed lines are hydrogen bonds. The figure was prepared with PyMol.

It filled the active site by interaction with amino acid residues located in the binding cavity, such as VAL-91, LEU-94, TRP-100, PHE-110, VAL-115, ALA-122, ILE-184, ASP-114, and HIS-393. In the conformation of the D_2_R–naringenin complex, oxygen atoms on the C=O functional group at the C-4 bond site formed good hydrogen bonds with the hydrogen-donor ASP-114 residue, and oxygen atoms in the C-7 functional group on the A ring formed stable hydrogen bonds with the donor HIS-393 residues. The lengths of hydrogen bonds were 3.1 Å and 3.2 Å, respectively. Hydrogen bonds remarkably enhanced the interaction and fastened the spatial conformation of the complex in the binding site. In addition, hydrophobic interactions were formed with VAL-91, LEU-94, TRP-100, PHE-110, VAL-115, ALA-122, ILE-184, PHE-189, VAL-190, PHE-198, PHE-382, TRP-386, PHE-389, PHE-390, and TRP-413, as shown in Figure 8. This molecular simulation suggests that naringenin is a potential D_2_R antagonist, although the results did not definitively resolve these hypotheses in an experimental test. These results provide the initial underpinnings for molecularly-derived models of gastrointestinal-promoted drug actions at D_2_Rs and give a precursor for the synthesis of target drugs with high efficiency and low toxicity.

## 3. Materials and Methods

### 3.1. Materials and Reagents

Dried Aurantii fructus (Sample No. 20170716) was collected from Yuanjiang City, Hunan Province and identified by Aihua Liu (senior engineer of Hunan Hansen Pharmaceutical Co., Ltd., Yiyang, China). Organic solvents including *n*-hexane, ethyl acetate, *n*-butanol, methanol, ethanol, acetic acid, and deionized water were used for sample preparation and isolation; analytical-grade acetonitrile was used for high performance liquid chromatography (HPLC) analysis (Sinopharm Chemical Reagent Co., Ltd., Shanghai, China); and ultra-pure water was obtained from a hyperpure water purifier (Barnstead Easy Pure II, Merck chemical technology, Co. Ltd., Shanghai, China). D101 macroporous resin was purchased from Cangzhou Bon Adsorber Technology Co., Ltd. (Cangzhou, China). Any reagents or sample solutions were degassed and filtered before HSCCC or HPLC analysis.

### 3.2. Molecular Docking

To screen D_2_R antagonists and illustrate the pharmacological actions of AF, a molecular docking study capable of predicting the structure of a ligand within the constraints of a receptor binding site was employed. The 3D structure of the D_2_ dopamine receptor was obtained from the Protein Data Bank (ID: 6CM4) [23]. The original ligands and water molecules were deleted from the crystal structure of 6CM4 via PyMOL. The structures of bioactive compounds from AF were collected from the TCM database (as we reported previously in detail [2]) and were drawn using ChemBioDraw Ultra and ChemBio 3D Ultra. The docking simulation between ligands and 6CM4 was performed by AutoDock Vina [24]. The grid was concentrated on the binding site (40 × 40 × 40 Å^3^, 0.375 Å, central coordinates *x* = 9.177, *y* = 5.470, and *z* = −8.665) to screen the specific ligands of D_2_R. The docking score was calculated and repeated for each ligand in triplicate. PyMOL was performed to illustrate the interactions between receptor and ligand. Then, the active compounds with potential antagonistic ability on D_2_Rs were screened.

### 3.3. Sample Preparation

For sample preparation, 850 g AF powder (80 meshes) was extracted with 3.0 L 60%–80% ethanol in the dark at room temperature for 24 h with suitable stirring, and the filter liquor was collected. The filter residue was extracted again with the same conditions. The filtrates were gathered and concentrated by a rotary evaporator (R1001, Zituo instrument equipment Co., Ltd., Zhengzhou, China) under reduced pressure at 55 °C until there was no smell of ethanol, a 1000 mL concentrate of AF was obtained and the pH was adjusted to 2–3 using HCl. The concentrate was flowed at 2–3 BV/h through a column containing 500 mL D101 macroporous resin (2 × 40 cm) and kept for 30 min. This resin was washed to colorless (about 5 BV) and then eluted with 10 BV 70% ethanol and 10 BV 95% respectively with a flow rate of 3 BV/h. Then, 70% ethanol eluent and 95% ethanol eluent were concentrated respectively under a vacuum and freeze-drying apparatus (MODULYOD-230, Thermo Fisher Scientific, Waltham, MA, USA). The weights of 70% concentrated crude extract and 95% concentrated crude extract were 30.1 g and 4.2 g, respectively. The concentrated crude extracts were stored at −20 °C and prepared for the later HPLC detection and HSCCC separation.

### 3.4. HPLC Analysis

HPLC was performed by use of an LA-20AT HPLC system (Shimadzu Co., Ltd., Kyoto, Japan), equipped with a quatpump, a 20 μL sample loop, a thermostatic column regulator, a diode assay detector (DAD), and a ChemStation. Chromatographic separation was performed on a GL Science Wondasil^TM^ C18 column (4.6 mm × 250 mm, 5 μm). The mobile phase was composed of A (0.2% phosphoric acid in ultrapure water) and B (acetonitrile) with a program of gradient elution (0–45 min, 10–65% B) at a flow rate of 1.0 mL/min. The detected wavelength was set at 280 nm and the column temperature was maintained at 30 °C.

### 3.5. HSCCC Apparatus

The preparative HSCCC was employed by the use of a type TBE-300A HSCCC (Tauto Biotechnique Co., Ltd., Shanghai, China), consisting of an upright coil type-J planet centrifuge with three multilayered coils (diameter of PTFE tube = 1.6 mm, total volume = 260 mL) and a 20 mL manual sample loop. The system was also equipped with a DC-0506 low-temperature thermostatic bath (Shanghai Sunny Hengping Scientific Instrument Co., Ltd., Shanghai, China), a TBP-1002 medium-pressure constant flow pump, and a full-wavelength model TBD-500 UV detector (Shanghai Tauto Biotechnique Co., Ltd., Shanghai, China) and a WH V4.0 workstation (Shanghai Wuhao Information Technology Co., Ltd., Shanghai, China). The revolution speed of the speed controller could be regulated from 0 to 1000 rpm.

### 3.6. HSCCC Separations

*n*-Hexane−ethyl acetate−*n*-butanol−methanol—0.05% acetic acid (1:3:1.8:1:5, *v*/*v*/*v*/*v*/*v*) was prepared as a solvent system for the first-step HSCCC isolation that was carried out as follows: The upper phase (stationary phase) was pumped into the multilayer-coiled column at a speed of 30 mL/min. Then, the lower phase (mobile phase) was pumped into the multilayer-coiled column at a speed of 2.0 mL/min, the device was running at a speed of 900 rpm, and the temperature of the multilayer-coiled column was constant at 20 °C. As the sample solution, 150 mg of the crude extract of 70% ethanol eluent was dissolved in 20 mL of separated lower phase and filtered through a 0.45 μm membrane filter. When the hydrodynamic equilibrium of two phases was balanced and the retention rate of stationary phase was appropriate, the prepared solution was injected through the sample loop. Effluent was continuously detected at 280 nm and collected.

*n*-Hexane−*n*-butanol−ethanol (methanol)−0.05% acetic acid (2:0.6:1:3, *v*/*v*/*v*/*v*) solvent system was employed for the second step of HSCCC isolation, and the other conditions were identical to the first step of the HSCCC isolation mentioned above, except that 120 mg crude extract of 95% ethanol eluent was prepared as the sample solution in 20 mL lower phase. Each peak of HSCCC was collected based on the chromatographic indication, the collected fractions were evaporated, freeze-dried, and analyzed by HPLC.

### 3.7. Structure Identification

The purified compounds from AF were identified by mass spectrometry (6530 ESI-Q-TOF-MS) with an Agilent 1290/MSD (Agilent, Santa Clara, CA, USA), and NMR spectra (^1^H-NMR, and ^13^C-NMR) were obtained with a Bruker Avance 500 spectrometer with tetramethylsilane (TMS) as internal standard (Analysis and Testing Center of Chemical Research Institute of Hunan Normal University).

## 4. Conclusions

In the present work, a bioactivity-guided method based on molecular docking combined with effective two-step HSCCC procedures was established for the successful separation of potential D_2_R antagonists with high purities from the ethanolic extract of AF. *n*-Hexane−ethyl acetate−*n*-butanol−methanol−0.05% acetic acid (1:3:1.8:1:5, *v*/*v*/*v*/*v*/*v*) and *n*-hexane−*n*-butanol −ethanol (methanol)−0.05% acetic acid (2:0.6:1:3, *v*/*v*/*v*/*v*) solvent systems were successfully applied to isolate the target compounds, including naringin (**1**), neohesperidin (**2**), meranzin (**3**), poncirin (**4**), meranzin hydrate (**5**), isomeranzin (**6**), nobiletin (**7**), and tangeretin (**8**), with high purities over 92.4%. The antagonistic abilities of these basic compounds and their metabolites on D_2_R were investigated based on a molecular docking study. Naringenin, a basic structure of naringin with a high content in AF, was demonstrated as an excellent D_2_R antagonist. The docking results provided the initial underpinnings for molecularly-derived models of gastrointestinal-promoted drug actions at D2Rs and supported the argument that the excellent antagonists with high yields could be chemical markers of Aurantii fructus. This study provides a feasible method to screen and isolate gastrointestinal motility drugs from natural sources. The pharmacological mechanism of AF for the treatment of gastrointestinal motility disorder is clearly illustrated on a molecular level in silico.

## Figures and Tables

**Table 1:** Screened potential antagonists of D_2_ dopamine receptor (D_2_R) from Aurantii fructus (AF) by the use of docking score, potential prediction, and feasibility of preparative separation.

Compound	Docking Score (kcal/mol)	Potential Prediction ^a^	Feasibility of Separation ^b^
**Flavonoids**			
Diosmetin-7-*O*-glucoside	−9.3	√	
Apigenin	−9.1	√	
Eriocitrin	−5.5		√
Neoeriocitrin	−5.3		√
Narirutin	−5.5		√
Naringin ^c^	−5.4		√
Hesperidin	−2.8		√
Neohesperidin	−3.4		√
Poncirin	−3.2		√
Naringenin	−9.3	√	√
Nobiletin	−8.0		√
5,6,7,4′-Tetramethoxyflavone	−8.8	√	
Tangeretin	−8.6	√	√
**Coumarins**			
Meranzin hydrate	−8.6	√	√
Meranzin	−7.1		√
Epoxybergamottin	−9.2	√	
Isomeranzin	−7.4		√
Auraptene	−9.1	√	
*β*-sitosterol ^d^	−9.0	√	

^a^ The docking score of the positive drug (domperidone) was −10.7 kcal/mol, so the score of candidate compounds should be less than −8.5 kcal/mol for further separation according to drug screening principles. ^b^ The feasibility of preparative separation of each compound was preliminarily analyzed using the results of our previous study and related literature data. ^c^ Before entering the body, flavonoid glycosides of AF are hydrolyzed to metabolites in the gastrointestinal tract and then absorbed for their corresponding pharmacological effect. Therefore, as the flavonoid glycoside of the best score compound (naringenin), naringin should be classed as the candidate compound for preparative separation. ^d^ the compound do not belong to coumarins.

**Table 2:** *K*-values of the compounds in different two-phase solvent systems of the first-step high-speed counter-current chromatography (HSCCC) separation.

No.	Solvent Systems	*K*1	*K*2	*K*3	*K*4
1	*n*-hexane: ethyl acetate: *n*-butanol: methanol: 0.05% acetic acid water = 1:1:1:1:3	0.37	0.34	2.86	0.90
2	*n*-hexane: ethyl acetate: *n*-butanol: methanol: 0.05% acetic acid water = 1:3:2:1:5	1.47	0.94	7.30	3.35
3	*n*-hexane: ethyl acetate: *n*-butanol: methanol: 0.05% acetic acid water = 1:3:1.8:1:5	1.01	0.62	3.80	2.53
4	*n*-hexane: ethyl acetate: *n*-butanol: ethanol: 0.05% acetic acid water = 1:3:1.8:1:5	1.47	0.85	4.70	3.60

**Table 3:** *K*-values of the compounds in different two-phase solvent systems of the second step of high-speed counter-current chromatography separation.

No.	Solvent Systems	*K5*	*K6*	*K7*	*K8*
5	*n*-hexane: ethyl acetate: methanol: 0.05% acetic acid water = 2:1:1:3	4.17	5.77	4.57	5.54
6	*n*-hexane: ethyl acetate: *n*-butanol: methanol: 0.05% acetic acid water = 2: 0.5:0.5:1:3	1.56	2.33	2.01	6.38
7	*n*-hexane: *n*-butanol: ethanol: 0.05% acetic acid water = 2: 0.6:1:3	1.03	1.95	1.36	5.58
8	*n*-hexane: *n*-butanol: methanol: 0.05% acetic acid water = 2: 0.6:1:3	0.90	1.78	1.28	5.08

**Table 4:** Screening of potential D_2_R antagonists from typical compounds of Aurantii fructus.

Compound	Score (kcal/mol)	Hydrophobic Residues (within a range of 4 Å)	H-Bond	Hydrogen Bond Residue
Risperidone ^a^	−12.0	TRP-100, PHE-110, VAL-115, ALA-122, PHE-198, PHE-382, TRP-386, PHE-389, PHE-390	1	ASP-114
Domperidone ^b^	−10.7	VAL-91, LEU-94, TRP-100, PHE-110, VAL-115, ALA-122, ILE-184, PHE-189, PHE-198, PHE-382, TRP-386, PHE-389, PHE-390, TRP-413	1	ASP-114
Naringenin	−9.3	VAL-91, LEU-94, TRP-100, PHE-110, VAL-115, ALA-122, ILE-184, PHE-189, VAL-190, PHE-198, PHE-382, TRP-386, PHE-389, PHE-390, TRP-413,	2	ASP-114, HIS-393

^a^ original ligand; ^b^ positive drug.

## Supplementary Materials

Table S1. Docking score of antagonistic ability on D_2_ dopamine receptor, potential prediction and feasibility of preparative separation of chemical compounds from Aurantii fructus; Figure S1. The detailed data of isolated compounds with high purity identified by ESI-Q-TOF-MS, 1H-NMR and 13C-NMR.
