# Supplementary material for: Bioactivity-Guided Separation of Potential D2 Dopamine Receptor Antagonists from Aurantii Fructus based on Molecular Docking Combined with High-Speed Counter-Current Chromatography
Source: Molecules. 2018 Nov 29;23(12):3135. doi: 10.3390/molecules23123135 (PMC6320876; doi:10.3390/molecules23123135)
Supplement: Supplementary file 1 [file molecules-23-03135-s001.pdf]

Supplementary material

Tab.S1. Docking score of antagonistic ability on D_2 dopamine receptor, potential prediction and feasibility of preparative separation of chemical compounds from *Aurantii fructus*

No.	Compound	Docking score (kcal/mol)	Potential prediction [a]	Feasibility of preparative separation [b]
	**Alkaloids**			
14	Aminobenzoic acid	-6.3		
15	Pipecolic acid	-5.9		
16	Piperidineacetic Acid	-5.8		
17	Synephrine	-6.3		
18	*N*-methyltyramine	-6.5		
19	Adenosine	-7.1		
20	*N*-Acetylnorsynephrine	-7.1		
	**Flavonoids**			
39	Quercetin-3-*O*-rutinoside (Rutin)	-5.0		
40	Isovitexin-7-*O*-glucoside (Saponarin)	-4.7		
41	Diosmetin-6,8-*di*-*C*-glucoside	0.1		
42	Diosmetin-6,8-*di*-*C*-glucoside isomer	0.1		
43	Apigenin-7-*O*-rutinoside-4'-*O*-glucoside (Isorhoifolin-4'-*O*-glucoside)	-3.3		
44	Diosmetin-7-*O*-glucoside	-9.3	√	
45	Luteolin-7-*O*-rutinoside (Veronicastroside)	-7.5		
46	Apigenin-7-*O*-rutinoside (Isorhoifolin)	-7.8		
47	Diosmetin-7-*O*-rutinoside (Diosmin)	-8.0		
48	Diosmetin-7-*O*-neohesperidoside (Neodiosmin)	-6.6		
49	Diosmetin-7-O-(6''-*O*-acetyl) neohesperidoside	/		
50	Apigenin-7-*O*-neohesperidoside (Rhoifolin)	-7.9		
51	Isovitexin-7-*O*-xylocoside-2''-*O*-arabinoside	/		
52	Apigenin	-9.1	√	
	**Flavonones**			
21	Naringenin -7-*O*-triglycoside	/		

22	Eriodictyol-7-*O*-rutinoside (Eriocitrin)	-5.5		√
23	Eriodictyol-7-*O*-glucoside [c]	-7.3		
24	Eriodictyol-7-*O*-neohesperidoside (Neoeriocitrin)	-5.3		√
25	Naringenin-7-O-rutinoside (Narirutin)	-5.5		√
26	Naringenin-7-*O*-neohesperidoside (Naringin) [c]	-5.4		√
27	Naringenin-7-*O*-glucoside	-7.2		
28	Brutieridin	-1.6		
29	Hesperetin-7-O-rutinoside (Hesperidin)	-2.8		√
30	Hesperetin-7-*O*-neohesperidoside (Neohesperidin)	-3.4		√
31	Hesperetin-7-*O*-glucoside	-7.2		
32	Eriodictyol	-6.7		
33	Isosakuranetin-7-*O*-neohesperidoside (Poncirin)	-3.2		√
34	Isosakuranetin-7-*O*-glucoside (Isosakuranin)	-7.4		
35	Naringenin	-9.3	√	√
36	Melitidin	-2.9		
37	Hesperetin	-7.0		
38	Isosakuranetin	-6.9		
	**Polymethoxyflavonoids**			
53	3-Hydroxy-5,7,8-trimethoxyflavone	-7.7		
54	5-Hydroxy-6,7,3',4'-tetramethoxyflavone	-8.1		
55	5-Hydroxy-6,7,3',4',5'-pentamethoxyflavone	-7.7		
56	5,7,8,3',4'-Pentamethoxyflavone (Isosinensetin)	-7.5		
57	5,6,7,3',4'-Pentamethoxyflavone (Sinensetin)	-8.2		
58	5-Hydroxy-6,7,8,3',4'-pentamethoxyflavone (Demethylnobiletin)	-7.3		
59	5,6,7,8,3',4'-Hexamethoxyflavone (Nobiletin)	-8.0		√
60	3,5,6,7,8,3',4'-Heptamethoxyflavone	-6.7		
61	5,6,7,4'-Tetramethoxyflavone	-8.8	√	
62	5-Hydroxy-3,6,7,8,3',4'-hexamethoxyflavone	-7.0		
63	5-Hydroxy-3,6,7,8-tetramethoxyflavone	-6.9		
64	5,7,8,4'-Tetramethoxyflavone	-8.5		
65	5, 6, 7, 8, 4'-Pentamethoxyflavone (Tangeretin)	-8.6	√	√
66	3-Hydroxy-5,6,7,8,3',4'-hexamethoxyflavone (Natsudaidai)	-7.3		
67	5-Hydroxy-6,7,8,4'-tetramethoxyflavone	-8.0		

	**Triterpenes**			
68	Limonin	-3.0		
69	Limonin-17-*β*-*D*-glucoside	-4.3		
70	Nominin-17-*β*-*D*-glucoside	2.4		
71	Obacunoic acid-17-*β*-*D*-glucoside	0.2		
72	Nomilinic acid-17-*β*-*D*-glucoside	0.0		
73	Limonin isomer	/		
	**Coumarins**			
	Meranzin hydrate	-8.6	√	√
74	Phellopterin	-7.5		
75	Meranzin	-7.1		√
76	Xanthotoxol	-8.3		
77	Oxypeucedanin	-7.9		
78	Scopoletin	-7.3		
79	Epoxybergamottin	-9.2	√	
80	Isomeranzin	-7.4		√
81	Osthol	-7.4		
82	Auraptene	-9.1	√	
	**Others**			
83	Citric acid	-5.5		
84	5-Hydroxymethyl furfual	-5.1		
85	Paeoniflorin	-6.1		
86	Magnoloside A	-7.0		
	**TCM database**			
	p-Ocimene	-6.4		
	Myrcene	-6.2		
	Marmin	-8.4		
	β-sitosterol	-9.0	√	
	8_isopropylidene_1_5_dimethylcyclodeca_1_5_diene	-7.3		
	ZINC05224268	-6.9		
	**Essential oils**			
87	*P*-Xylene	-6.0		

88	(-)-α-Pinene	-6.1
89	α-Phellandrene	-7.1
90	3-carene	-6.3
91	Benzene	-4.9
92	*D*-limonene	-6.8
93	Ocimene	-6.4
94	4-carene	-6.3
95	Cyclohexene	-4.9
96	Linalool	-6.5
97	Terpineol	-7.2
98	Thymol	-7.1
99	Limonene oxide	-6.7
100	Copaene	-6.9
101	1.6-Cyclodecadiene	/
102	Naphthalene	-7.4
103	(+)-Aromadendrene	-7.4
104	2-Naphthalenemethanol	-7.9

[a] The docking score of the positive drug (domperidone) was 10.7 kcal/mol, so the score of candidate compounds should be less than -8.5 kcal/mol for the further separation according to drug screening principles;

[b] The feasibility of preparative separation of each compound was preliminarily analyzed by our previous study and related literature data.

[c] Before entering the body, flavonoid glycosides of AF will be hydrolyzed to metabolites in the gastrointestinal tract and then be absorbed for corresponding pharmacological effect. Therefore, as the flavonoid glycoside of naringenin, naringin should be classed to the candidate compound for preparative separation.

Fig.S1. The detailed data of isolated compounds with high purity identified by ESI-Q-TOF-MS, 1H-NMR and 13C-NMR.

Compound 1: naringin

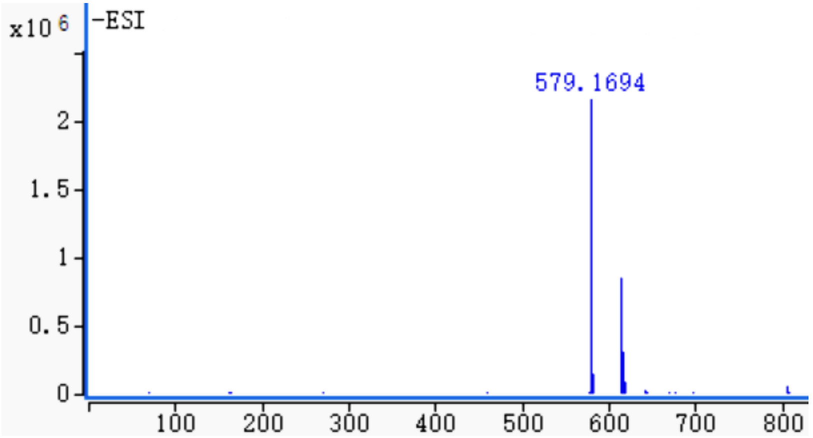

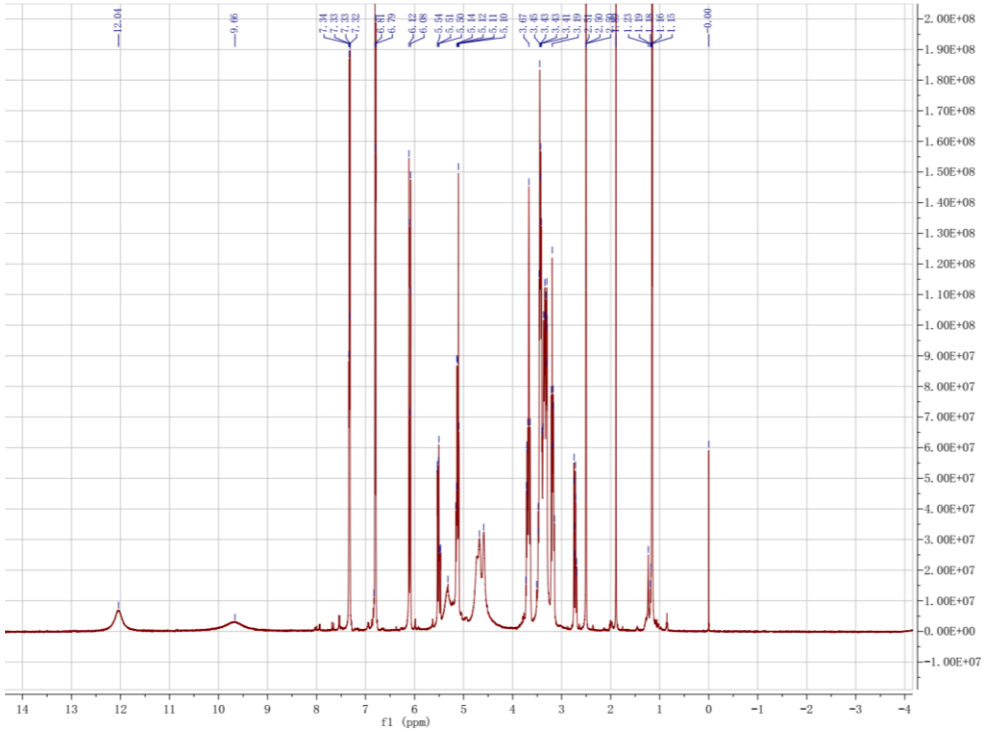

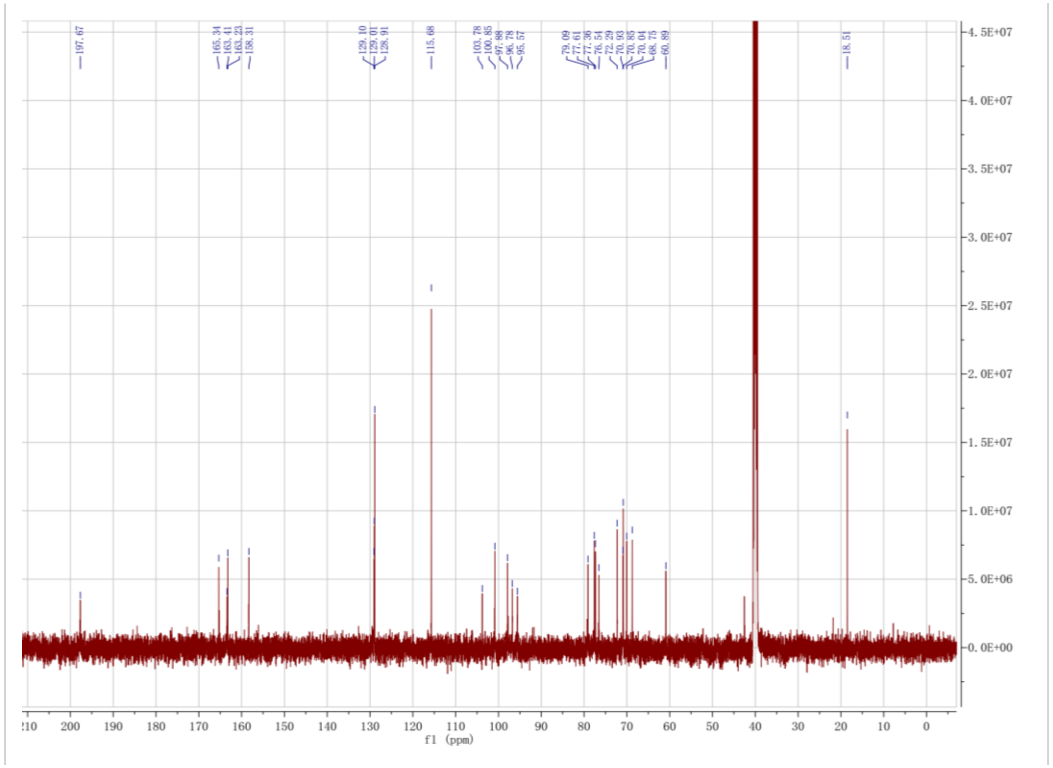

Compound 2: neohesperidin

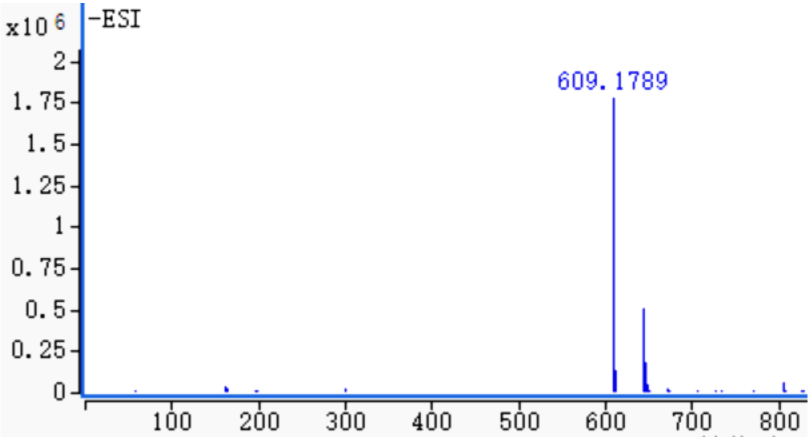

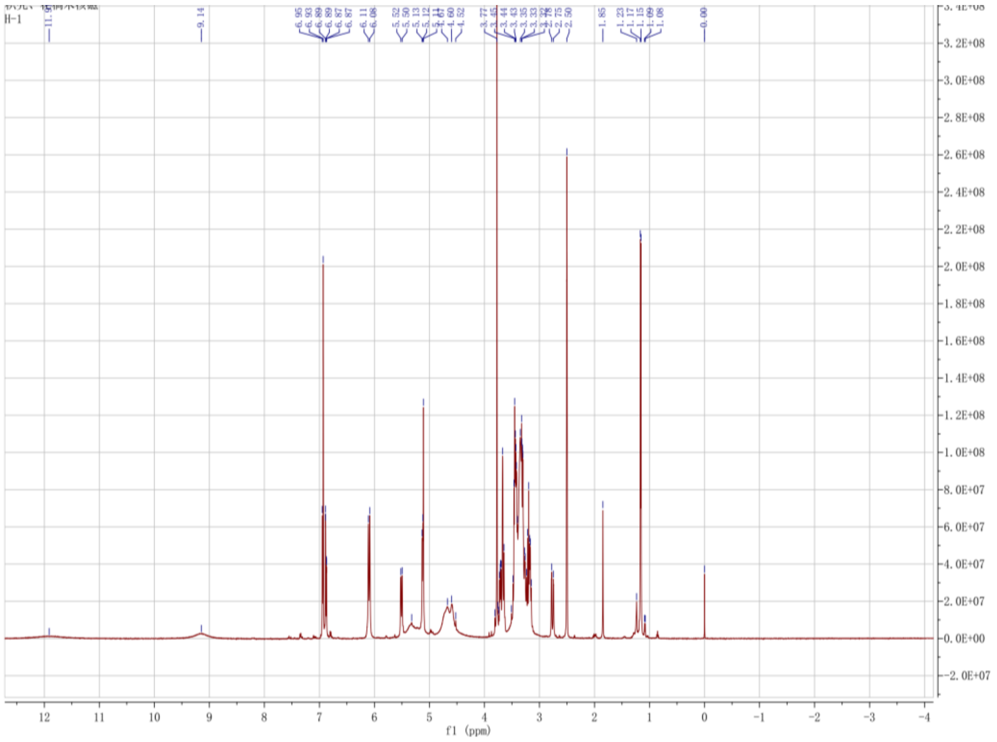

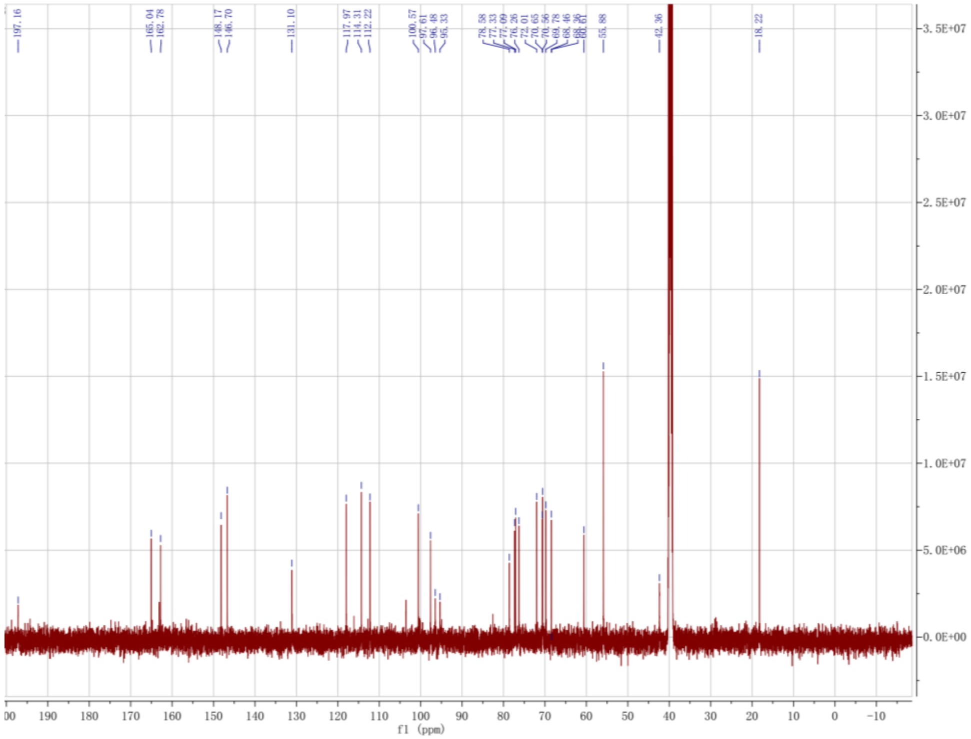

Compound 3: meranzin

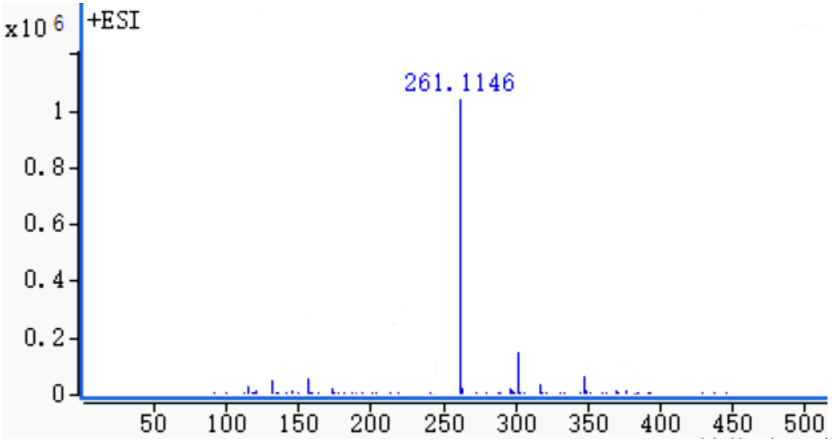

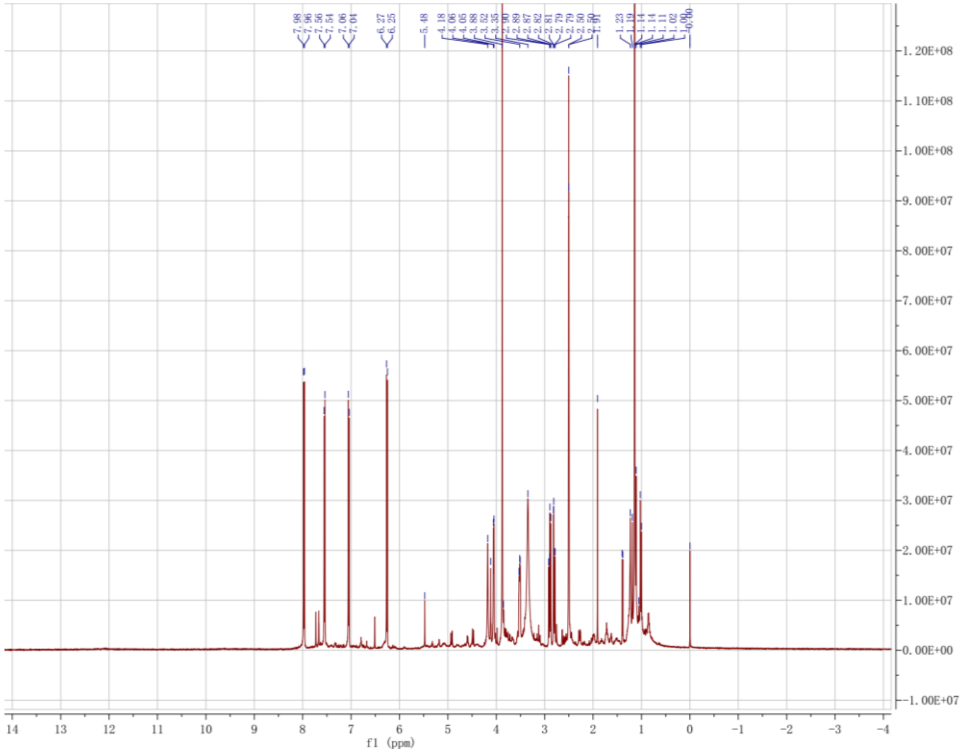

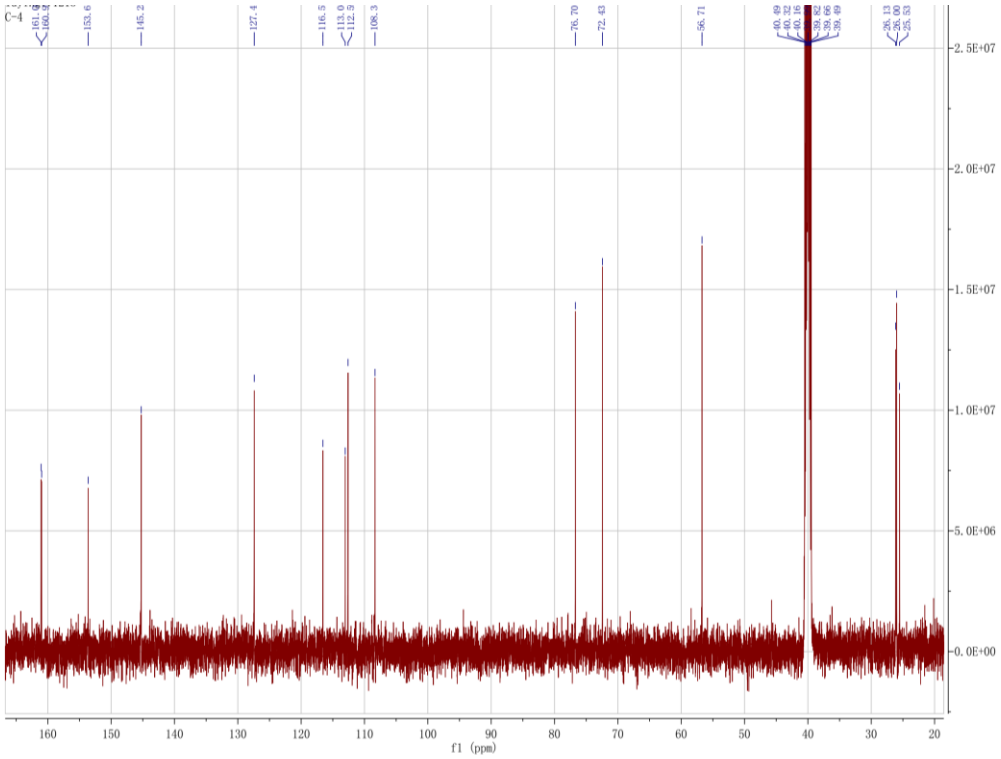

Compound 4: poncirin

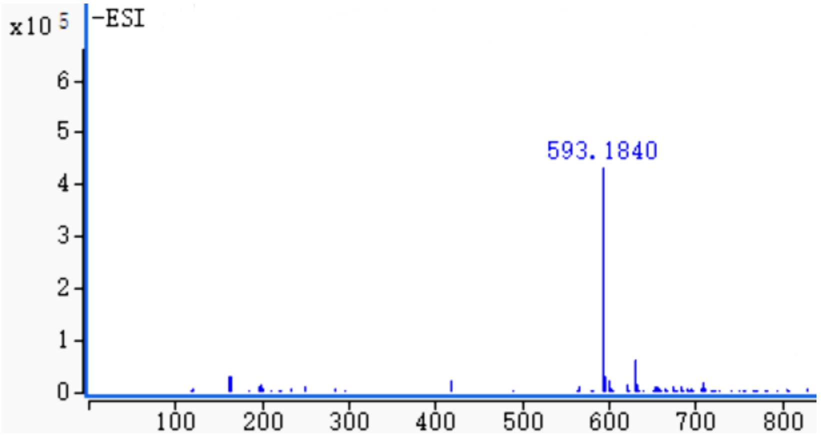

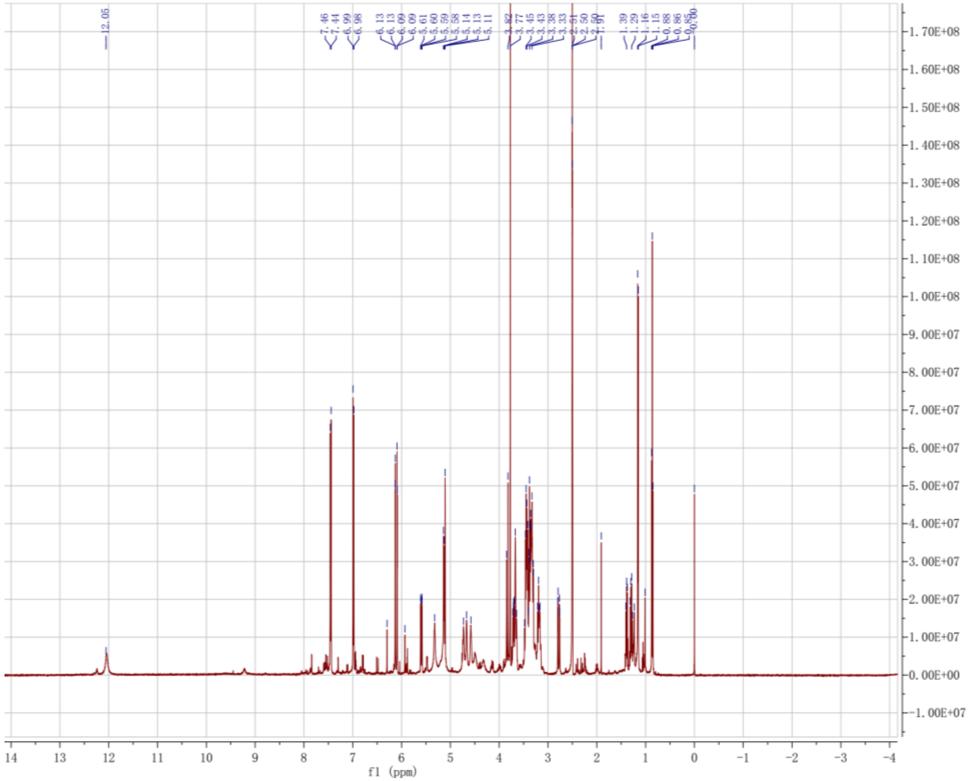

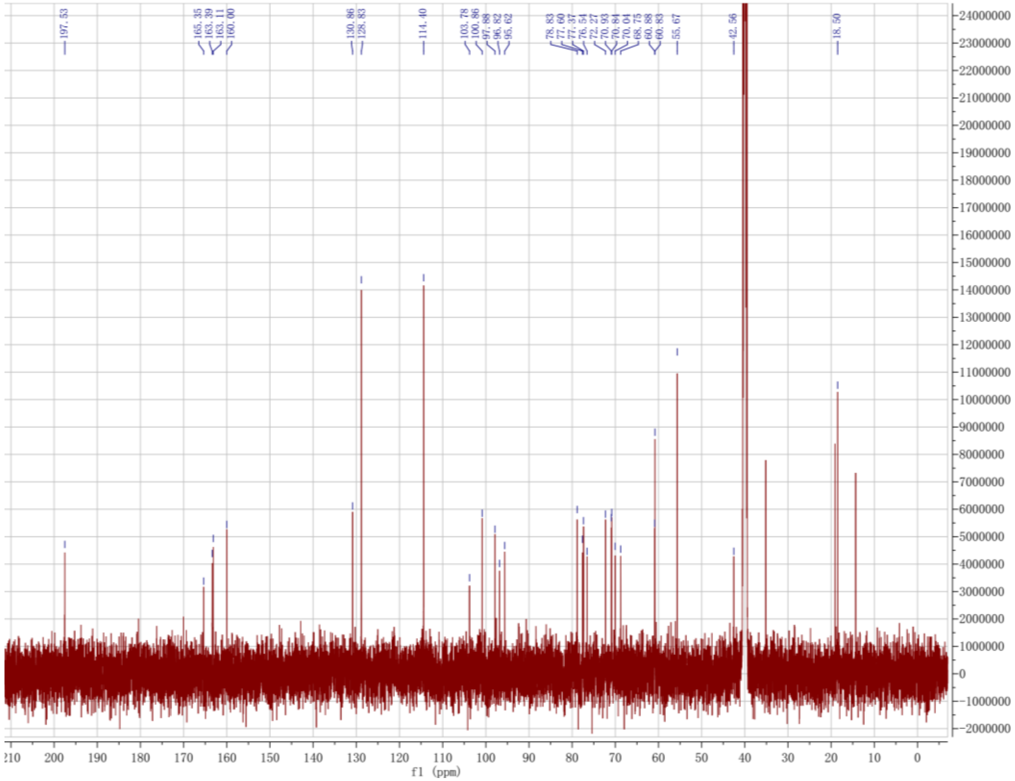

Compound 5: meranzin hydrate

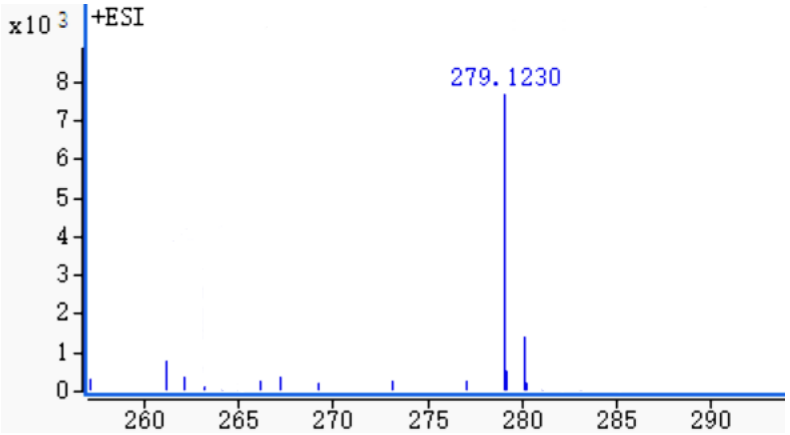

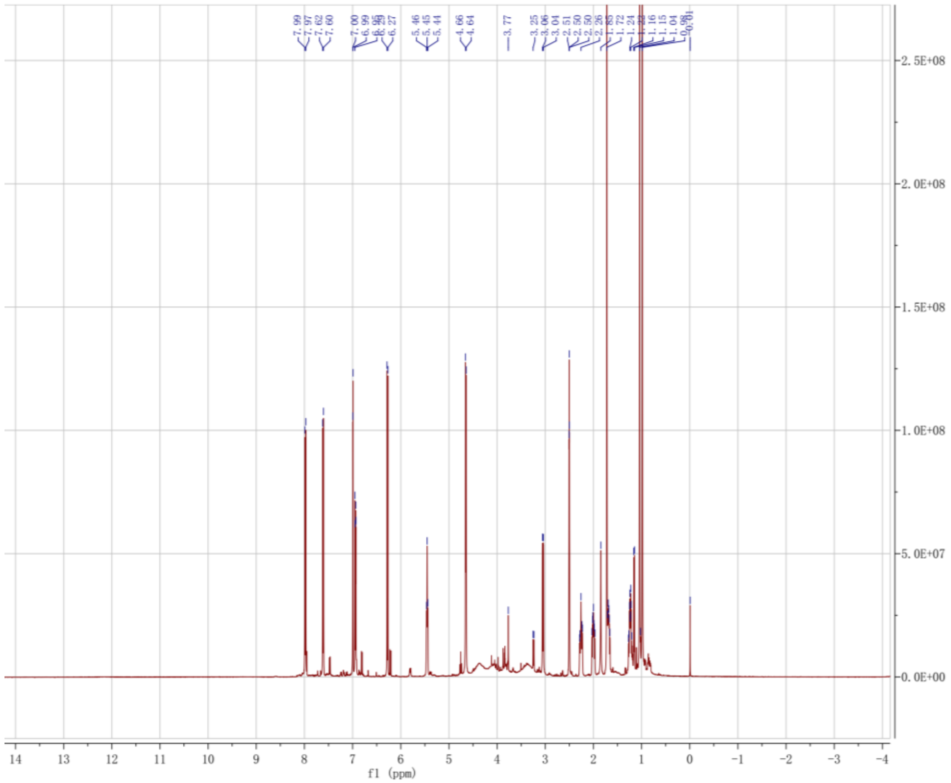

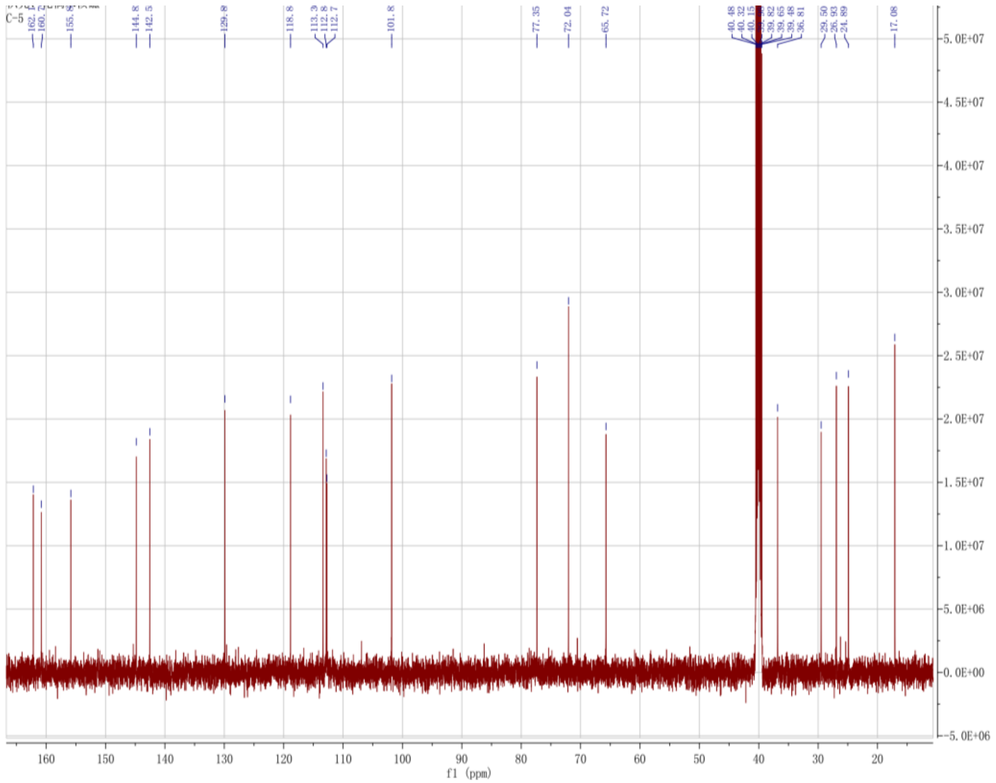

Compound 6: isomeranzin

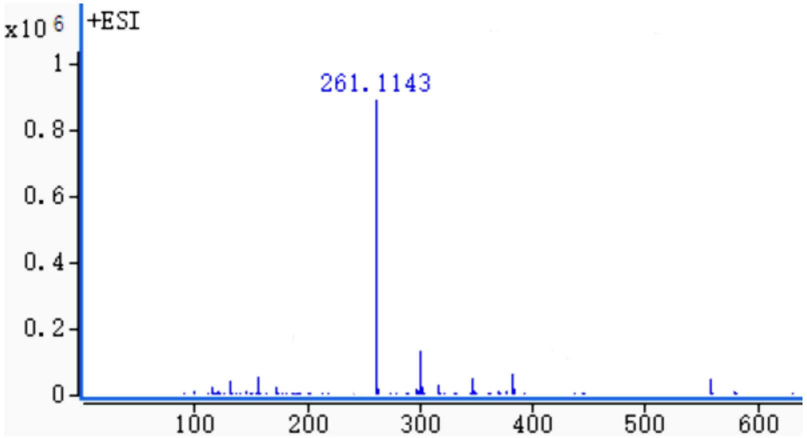

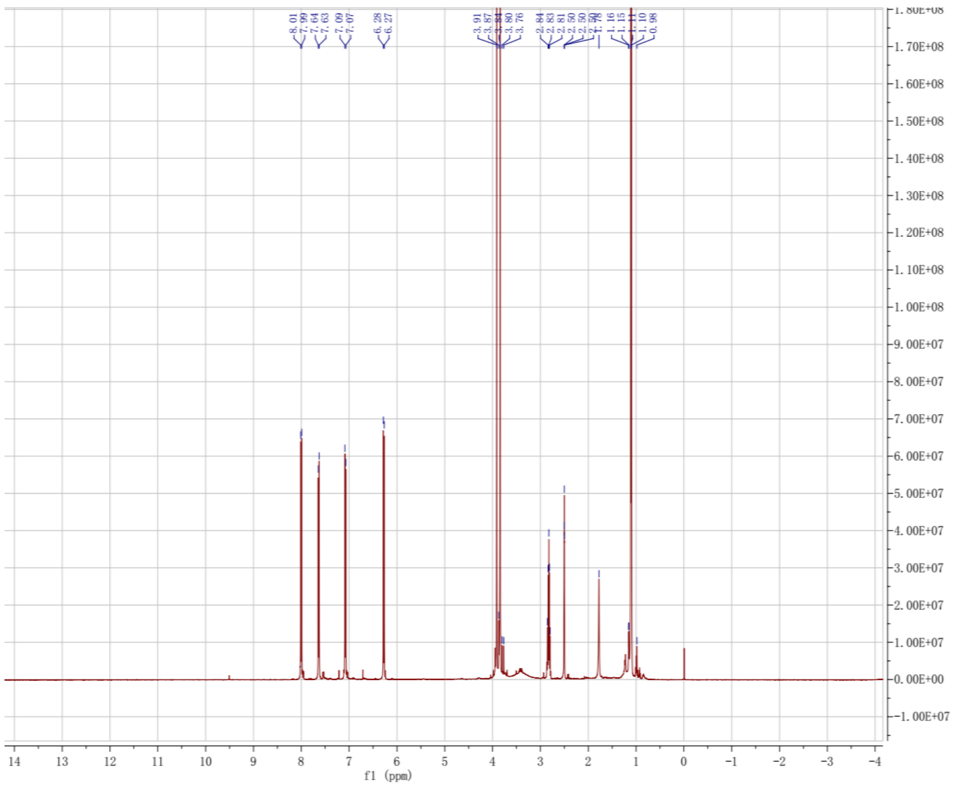

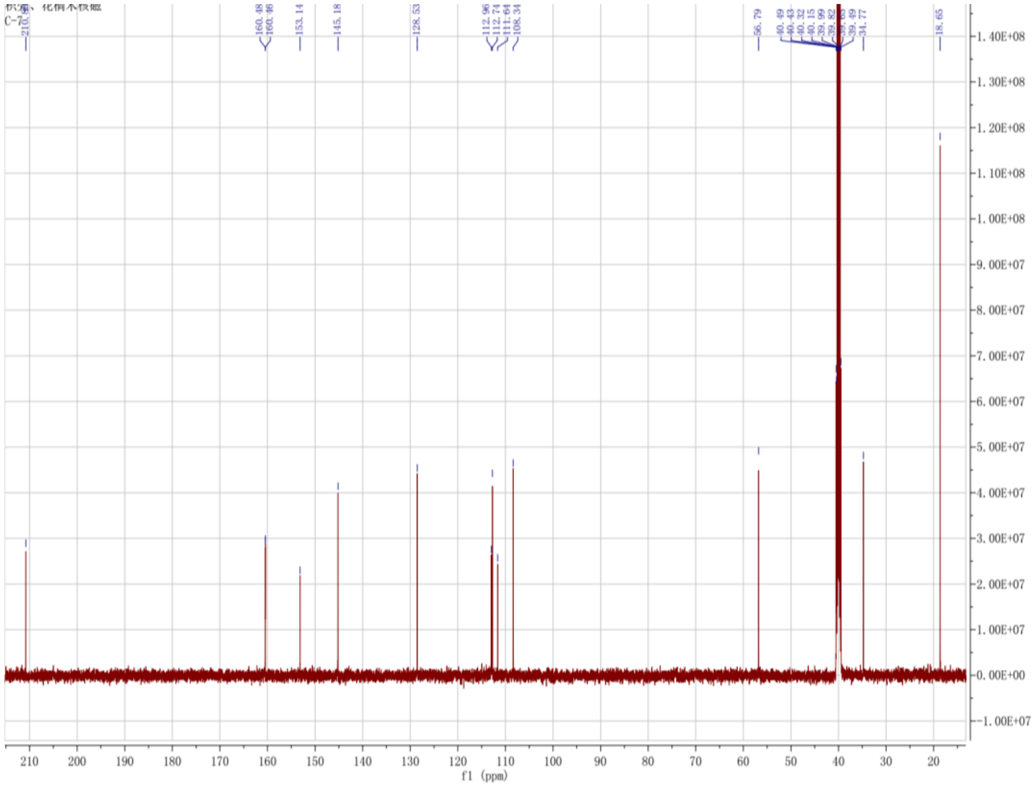

Compound 7: nobiletin

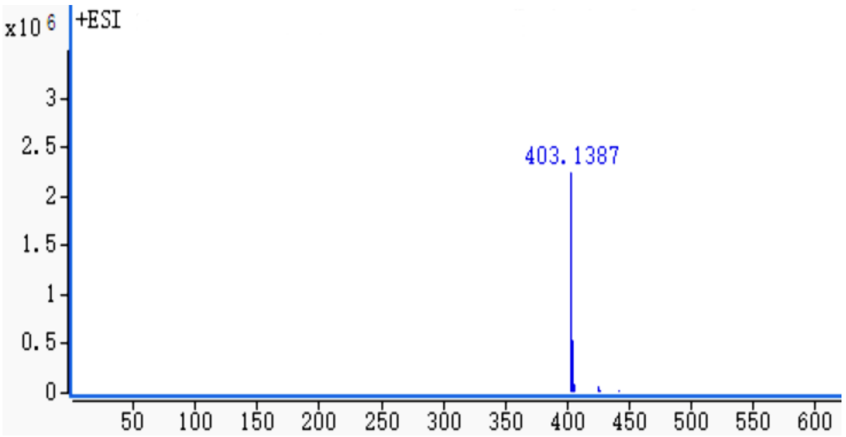

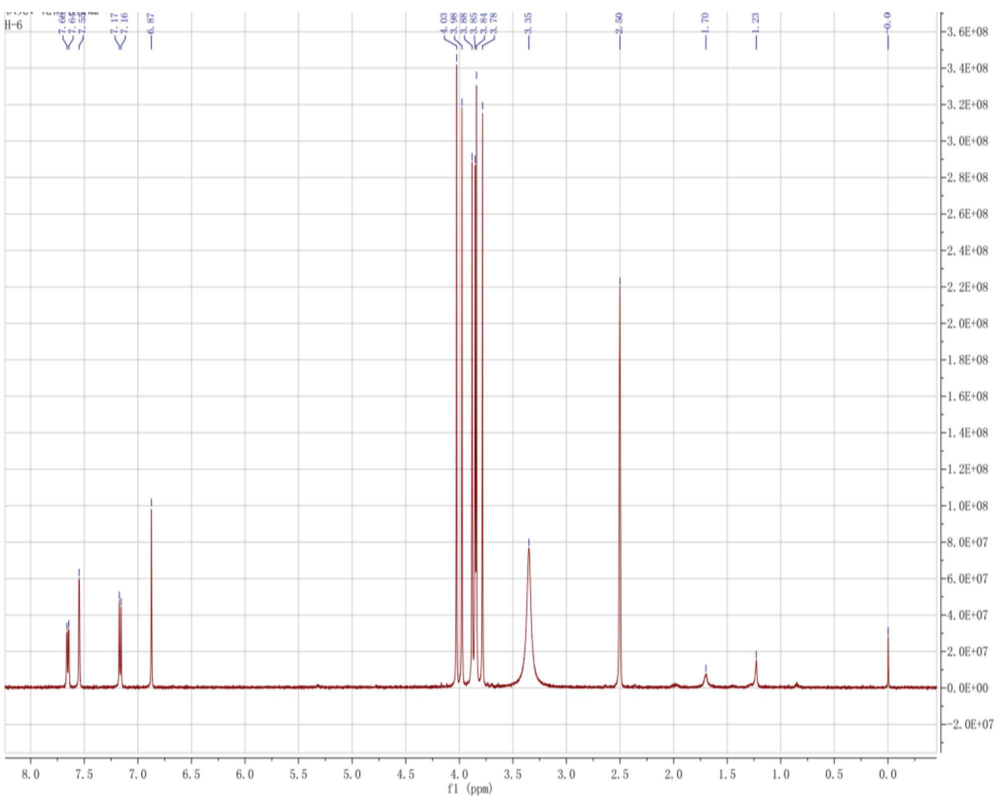

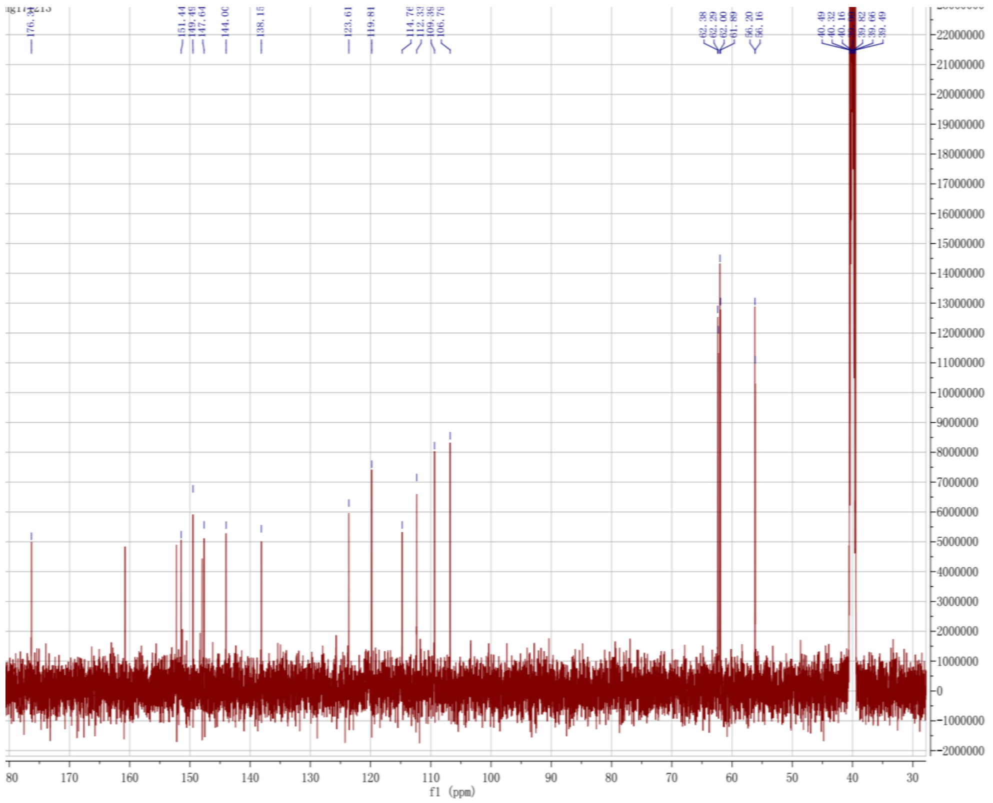

Compound 8: tangeretin

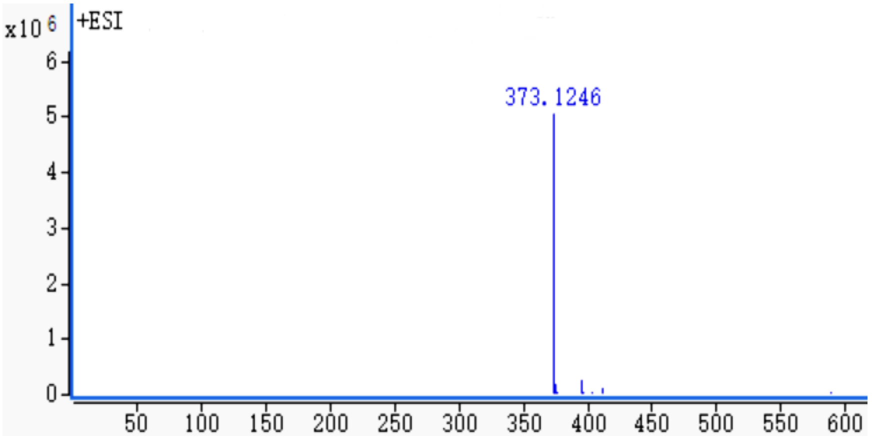

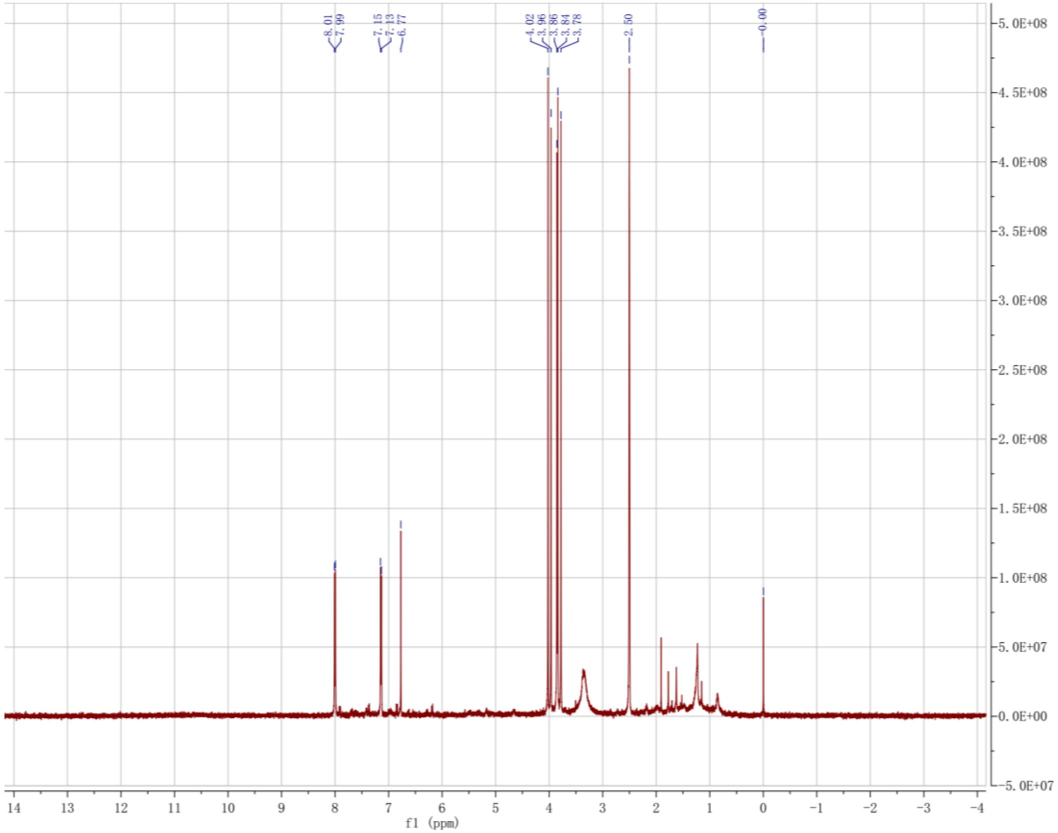

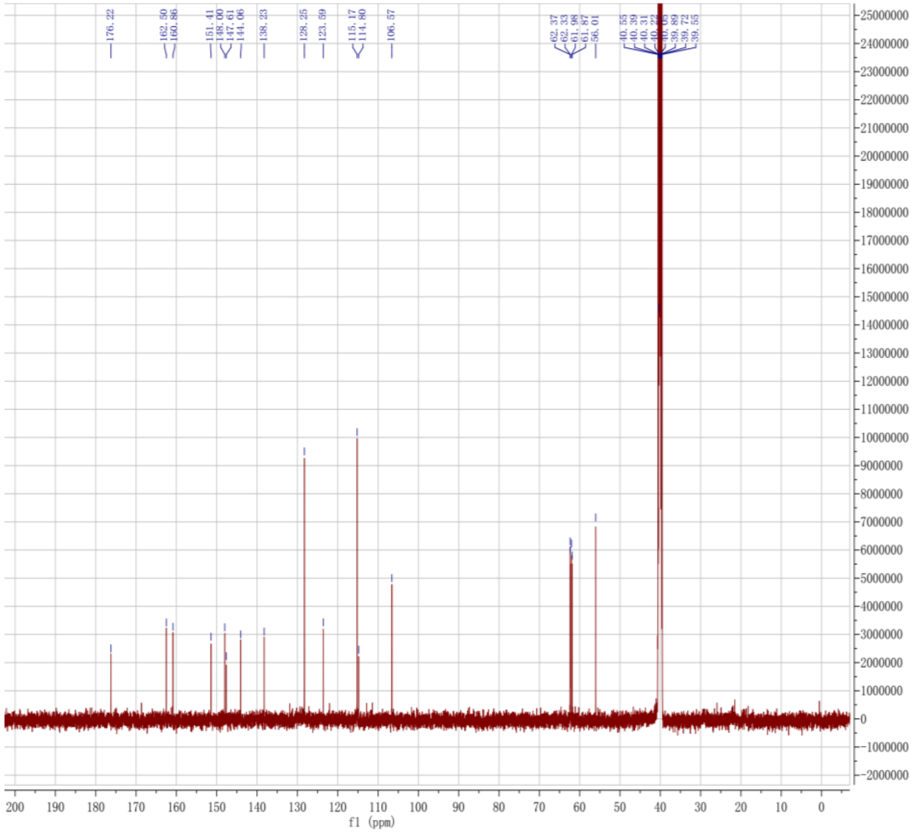

Compound 1: deprotonated molecule at m/z 579.1694 $[M - H]^-$ with chemical formula C27H32O14; 1H-NMR (500 MHz, DMSO-d6) δ-H: 12.04 (1H, s, 5-0H), 9.66 (1H, s, 4′-OH), 7.33 (2H, dd, J=8.5, 4.0Hz, H-2′, 6′), 6.80(2H, d, J=8.5, Hz, H-3′, 5′),6.12(1H, d, J=2.0 Hz, H- 8), 6.08(1H, t, J=6.0 Hz, H-6), 5.51(1H, m, H- 1′′), 5.12(1H, m, H- 1′′′), 3.18(1H, m, H- 3), 2.72(1H, m, H- 6′′′), 3.67(3H, m), 3.45(4H, m), 3.35(1H, m, H-3α), 3.20(1H, m), 4.68(1H, s, OH), 4.59(1H, OH); 13 C-NMR(DMSO-d6) δ：79.09(C-2), 42.33(C-3), 197.67(C-4), 163.41(C-5), 96.78(C-6), 165.34(C-7), 95.57(C-8), 163.23(C-9), 103.78(C-10), 129.10(C-1′), 129.01(C-2′), 115.54(C-3′), 158.31(C-4′), 115.68(C-5′), 128.91(C-6′), 97.88(C-1′′), 77.61(C-2′′), 77.36(C-3′′), 69.79(C-4′′), 76.54(C-5′′), 60.89(C-6′′), 100.85 (C-1′′′), 70.85(C-2′′′), 70.93(C-3′′′),72.33(C-4′′′), 68.75(C-5′′′), 18.51(C-6′′′).

Compound 2: deprotonated molecule at m/z 609.1789 $[M - H]^-$ with chemical formula C28H34O15; 1H-NMR (500 MHz, DMSO-d6) δH:11.91(1H, s, 5-0H), 9.14 (1H, s, 3′-OH), 6.95(1H, s, H- 5′), 6.93(1H, s, H- 2′), 6.88(1H, dd, J=10.0,1.5 Hz, H-6′), 6.11(1H, s, H-6), 6.08(1H, s, H-8), 5.52 (1H, dd, J=4.0, 2.0 Hz, H-2), 5.33(1H,s, OH), 5.12 (1H, d, J=2.0 Hz, H- 1′′), 5.11(1H, s, H- 1′′′), 4.74(1H, s, OH), 4.60(1H, s, OH), 4.52(1H, OH), 3.77(3H, s, 4′-OCH_3), 3.67(3H, m), 3.45(4H, m), 3.35(1H, m, H-3α), 3.20(1H, m), 2.76 (1H, dd, J=4.0, 2.0 Hz, H-3β), 1.14 (3H, d, J=6.5 Hz, H- 6′′′); 13 C-NMR (DMSO-d6) δ: 78.58(C-2), 42.36(C-3), 197.16(C-4), 162.78(C-5), 96.48(C-6), 165.04(C-7), 95.33(C-8), 162.78(C-9), 117.97, 103.20(C-10), 131.10(C-1′), 112.22(C-2′), 146.70(C-3′), 148.17(C-4′), 114.31(C-5′), 100.57(C-1′′), 76.26(C-2′′), 77.33(C-3′′), 69.78(C-4′′), 77.09(C-5′′), 97.61(C-1′′′), 70.56(C-2′′′), 70.65(C-3′′′), 72.01(C-4′′′), 68.46(C-5′′′), 18.22(C-6′′′), 55.88(4′-OCH_3), 60.61(C-6′′).

Compound 3: protonated molecule at m/z 261.1146 $[M + H]^+$ with chemical formula C15H16O4; 1H- NMR(500 MHz, DMSO-d6)δ：7.97(1H, d, J=8.0 Hz , H-4), 7.55(1H, d, J= 8.5 Hz, H-5), 7.05(1H, d, J=8.5 Hz, H-6), 6.26(1H, d, J=9.5Hz, H-3), 3.51(1H, m, H-2′), 2.89(1H, dd, J=12.5, 2.0 Hz ,H-1′), 2.80(1H, dd, J=12.5, 2.0 Hz ,H-1′),3.88(3H, s, 7-OCH_3), 1.14(6H, d, J=3.0 Hz, 4′-CH_3, 5′-CH_3); 13C-NMR (DMSO-d6) δ：161.05(C-2), 160.97(C-7), 153.61(C-9), 145.25(C-4), 127.41(C-5), 108.34(C-8), 116.57(C-10),113.04(C-3), 112.59(C-6),56.71(OCH_3), 76.70(C-2′) ,72.43(C-3′), 26.13(C-1′), 26.00(3′-CH_3), 25.53(3′-CH_3).

Compound 4: deprotonated molecule at m/z 593.1840 $[M - H]^-$ with chemical formula C28H34O14; 1 H -NMR(500 MHz, DMSO-d6)δ：12.05(1H, s,5-OH), 7.45(2H, d, J=9.0 Hz , H-2′,6′), 6.98(2H, d, J=8.5 Hz , H-3′,5′), 6.13(1H, d, J=2.5 Hz , H-8), 6.09(1H, d, J=2.0 Hz, H-6), 5.59(1H, dd, J=12.5,2.0 Hz, H-2), 5.14(1H, d, J=7.0 Hz, H-1′′), 5.11(1H, s, J=9.5 Hz, H′′′), 3.77(3H, s, 4′-OCH_3), 2.78(1H, dd, J=12.5,3.5 Hz, H-3β), 1.15 (3H, d, J=6.5 Hz, H- 6′′′-CH_3). 3.26-3.40 (10H); 13C- NMR (125MHz, DMSO-d6) δ: 42.56(C-3),197.53(C-4), 165.35(C-7), 163.39(C-5), 163.11(C-9), 160.00(C-4′), 103.20(C-10), 130.86(C-2′, 6′), 128.83(C-1′),114.29(C-3′,5′), 100.78(C-1′′′),97.76(C-1′′), 96.90(C-6),95.62(C-8), 78.83(C-2), 77.42(C-5′′), 76.54(C-3′′), 72.27(C-2′′),70.93(C-2′′′), 70.84(C-4′′), 70.04(C-3′′′), 68.75(C-5′′′), 60.83(C-6′′), 18.5(C-6′′′), 55.67(4′- OCH_3).

Compound 5: protonated molecule at m/z 279.1230 $[M + H]^+$ with chemical formula C15H18O5; 1 H-NMR(500 MHz, DMSO-d6)δ：7.98(1H, d, J=9.5 Hz , H-4), 7.61(1H, d, J= 8.5 Hz, H-5), 6.95(1H, m, H-6), 6.27(1H, d, J=8.5Hz, H-3), 4.65(3H, d, J=9.6 , 7-OCH_3), 3.77(1H, m, -CH), 3.24(1H, d, J=2.0Hz OH), 3.50(1H, s, OH), 3.05(1H, d, J=13.2 Hz,1′-CH_2), 1.02(3H, s, 4′-CH_3), 0.92(3H, s, 5′-CH_3); 13 C-NMR (DMSO-d6)δ：162.17(C-2), 160.79(C-7), 155.85(C-9), 144.82(C-4), 129.89(C-5), 118.84(C-8), 113.36(C-8),112.84(C-3), 101.82(C-6), 77.35(C-2′), 71.95(C-3′), 29.50(C-1′), 26.93(C-4′), 24.89(C-5′).

Compound 6: protonated molecule at m/z 261.1143 $[M + H]^+$ with chemical formula C15H16O4; 1 H-NMR(500 MHz, DMSO-d6)δ：8.00(1H, d, J=9.0 Hz , H-4), 7.63(1H, d, J= 8.5 Hz, H-5), 7.08(1H, dd, J=2.5 Hz, H-6), 6.27(1H, d, J=9.5Hz, H-3), 3.91(1H, s, H-2′), 2.83(1H, m, H-1′), 3.91(3H, s, 7-OCH_3), 1.16(3H, s, 4′-CH_3), 1.11(3H, s, 5′-CH_3); 13C- NMR (DMSO-d6)δ: 210.72(C-2′), 160.48(C-2), 128.53(C-5), 112.96(C-8), 112.74(C-10), 111.64(C-3), 160.46(C-7), 153.14(C-9), 145.18(C-4), 108.34(C-6), 19.99 (C-4′, C-5′), 34.88(C-1′), 56.79(OCH_3).

Compound 7: protonated molecule at m/z 403.1387 $[M + H]^+$ with chemical formula C21H22O8; 1H -NMR(500 MHz, DMSO-d6) δ: 6.87(1H, s, H-3), 7.55(1H, d, J= 1.5 Hz,H-2′), 7.16(1H, d, J=8.5 Hz, H-5′), 7.65(1H, dd, J=8.5,1.5 Hz, H-6′),4.03(3H, s, OCH_3),3.98(3H, s, OCH_3), 3.88(3H, s, OCH_3), 3.85(3H, s, OCH_3), 3.84(3H, s, OCH_3), 3.78(3H, s, OCH_3); 13C-NMR(DMSO-d6) δ: 160.76(C-2), 106.28(C-3), 176.31(C-4), 144.00(C-5), 149.49(C-6), 138.01(C-7), 148.08(C-8), 147.64(C-9), 109.39(C-10), 123.61(C-1′), 111.33(C-2′), 152.15 (C-3′), 151.37(C-4′), 114.76(C-5′), 119.81(C-6′), 62.38(5-OCH_3), 62.29(6- OCH_3), 62.00(7- OCH_3), 61.89(8- OCH_3), 56.20(3′- OCH_3), 56.16(4′- OCH_3).

Compound 8: protonated molecule at m/z 373.1246 $[M + H]^+$ with chemical formula C20H20O7; 1H NMR(500 MHz, DMSO-d6)δ：6.77(1H, s, H-3), 8.00(2H, d, J= 3.5 Hz, H-2′, H-6′), 7.14(2H, d, J=5.1 Hz, H-3′, H-5′), 4.02(3H, s, OCH_3), 3.96(3H, s, OCH_3), 3.86(3H, s, OCH_3), 3.84(3H, s, OCH_3), 3.78(3H, s, OCH_3); 13C NMR (DMSO-d6) δ: 162.50(C-2), 106.57(C-3), 176.22(C-4), 144.06(C-5), 151.41(C-6), 138.23(C-7), 147.61(C-8), 128.25(C-2′, C-6′), 123.59(C-1′), 115.17(C-3′, C-5′), 114.80(C-10), 147.61 (C-9), 160.86(C-4′), 62.37(5- OCH_3), 62.33(6- OCH_3), 61.98(7- OCH_3), 61.87(8- OCH_3), 56.01(4′- OCH_3).
